# Association between severe acute pancreatitis and new-onset diabetes: a propensity score-matched real-world study

**DOI:** 10.3389/fendo.2025.1704688

**Published:** 2025-11-14

**Authors:** Djibril M. Ba, Phil A. Hart, Tian Qiu, Somashekar G. Krishna, Xiang Gao, Douglas L. Leslie, David Bradley, Jennifer Maranki, Kadiyatu Fofana, Vernon M. Chinchilli, Ariana R. Pichardo-Lowden

**Affiliations:** 1 Department of Public Health Sciences, Penn State College of Medicine, Hershey, PA, United States; 2 Division of Gastroenterology, Hepatology, and Nutrition, College of Medicine, The Ohio State University Wexner Medical Center, Columbus, OH, United States; 3 Department of Nutrition and Food Hygiene, School of Public Health, Institute of Nutrition, Fudan University, Shanghai, China; 4 Department of Medicine, Division of Endocrinology, Penn State College of Medicine, United States, Hershey, PA, United States

**Keywords:** AP, new-onset diabetes, real-world data, MarketScan, US

## Abstract

**Introduction:**

Acute pancreatitis (AP) is an inflammatory disease of the exocrine pancreas characterized by tissue damage and sometimes necrosis. However, whether severe AP is associated with an increased risk of incident diabetes remains unclear based on real-world data. This study aims to examine the relationship between severe AP and new-onset diabetes after hospitalization.

**Methods:**

We conducted a retrospective cohort study using the Merative™ MarketScan® claims database (2016–2023), identifying patients with AP and no prior history of diabetes at baseline. The exposure, severe AP, was defined by any of the following during hospitalization: pancreatic necrosis, hemodialysis, organ failure, or mechanical ventilation. We used multivariable stratified Cox proportional hazards regression models with propensity score strata to assess the association between severe AP and incident diabetes.

**Results:**

The matched study population consisted of 2,046 patients with severe AP and 2,046 patients with mild AP, with baseline characteristics well balanced between groups. Individuals with severe AP had a higher risk of developing diabetes compared with those with mild AP [adjusted hazard ratio (aHR) = 1.64, 95% confidence interval (CI): 1.30–2.06], after accounting for propensity score matching. The association between severe AP and incident diabetes was stronger in men (aHR = 2.03, 95% CI: 1.50–2.74) than in women (aHR = 1.06, 95% CI: 0.69–1.64; *P*-_interaction_ = 0.02).

**Discussion:**

In this large real-world data study, severe AP was associated with an increased risk of developing diabetes. These findings underscore the importance for glycemic surveillance and the need to consider proactive management of severe AP patients to mitigate their risk of poor health outcomes.

## Introduction

Acute pancreatitis (AP) is an inflammatory disease of the exocrine pancreas characterized by tissue damage and sometimes necrosis (1). AP is one of the most common gastrointestinal diseases requiring hospitalization in the United States (US) (2, 3). AP and diabetes often share a bidirectional relationship (4). Pancreatogenic diabetes (or type 3c diabetes) is an underdiagnosed form of secondary diabetes that occurs in the setting of a disease of the exocrine pancreas; AP is likely the most common cause (5, 6). It is likely the most common complication following an episode of AP and may develop more often than previously recognized, with a cumulative incidence ranging from 23% to 40% (7). Stress hyperglycemia is a common early feature in patients with AP. While the likelihood of persistent or worsening hyperglycemia is not well understood, in many cases, it is not a transient phenomenon (8), as patients with AP are at a higher risk of developing diabetes than those without AP. However, it remains unclear whether the severity of AP during hospitalization influences this risk. The revised Atlanta classification of AP is a clinically based categorization of AP severity into three degrees (mild, moderately severe, and severe), with severe AP being defined mainly by the presence of persistent systemic organ failure that is often accompanied by pancreatic necrosis (9). Previous research studies have frequently combined all cases of AP, regardless of severity during hospitalization, and their long-term influence on diabetes risk following discharge (10, 11).

Several epidemiological studies have reported the incidence of diabetes following an episode of AP. Two recent meta-analyses comprising 31 cohort studies indicated that approximately 23% of patients developed diabetes within 3 years following hospital discharge for AP (10, 11). However, many prior studies have been limited by small sample sizes, short follow-up, and lack of adjustment for confounders. Leveraging a large, real-world US claims database with longitudinal follow-up, the objective of this study was to investigate the association between severe AP during hospitalization and the risk and timing of new-onset diabetes following discharge. We hypothesized that, even after rigorous propensity score matching and adjustment for confounding variables, patients with severe AP would exhibit a significantly higher incidence and earlier onset of diabetes compared to those with mild AP. This study aims to provide more definitive evidence on this association by addressing key methodological limitations in the existing literature.

## Methods

### Study design

A retrospective cohort study with propensity score matching was conducted to examine the association between AP severity and the risk of new-onset diabetes following AP. All data were collected retrospectively. However, individuals with AP were followed prospectively in time to determine the risk of new-onset diabetes following discharge. The Penn State University Institutional Review Board considered this study not human participants’ research; thus, informed consent was not needed. This study followed the Strengthening the Reporting of Observational Studies in Epidemiology (STROBE) reporting guidelines for cohort studies (12).

### Data source

This study used the Merative™ MarketScan^®^ Commercial (MarketScan) database, a commercially available health insurance claims database. MarketScan is one of the largest and oldest nationwide longitudinal claims databases used for healthcare research, containing data on over 300 million unique, de-identified patients (13). The database consists of privately insured employees and their family members from large employers and health plans across all 50 US states and the District of Columbia (14). Longitudinal tracking of detailed patient-level healthcare claims information provides comprehensive data, including key demographic characteristics, healthcare utilization, inpatient and outpatient medical information with diagnosis codes, procedure codes, detailed prescription drugs, and financial information (15). MarketScan is fully compliant with the Health Insurance Portability and Accountability Act of 1996 (HIPAA) (15).

### Cohort derivation and assessment of exposure

**Figure 1:**
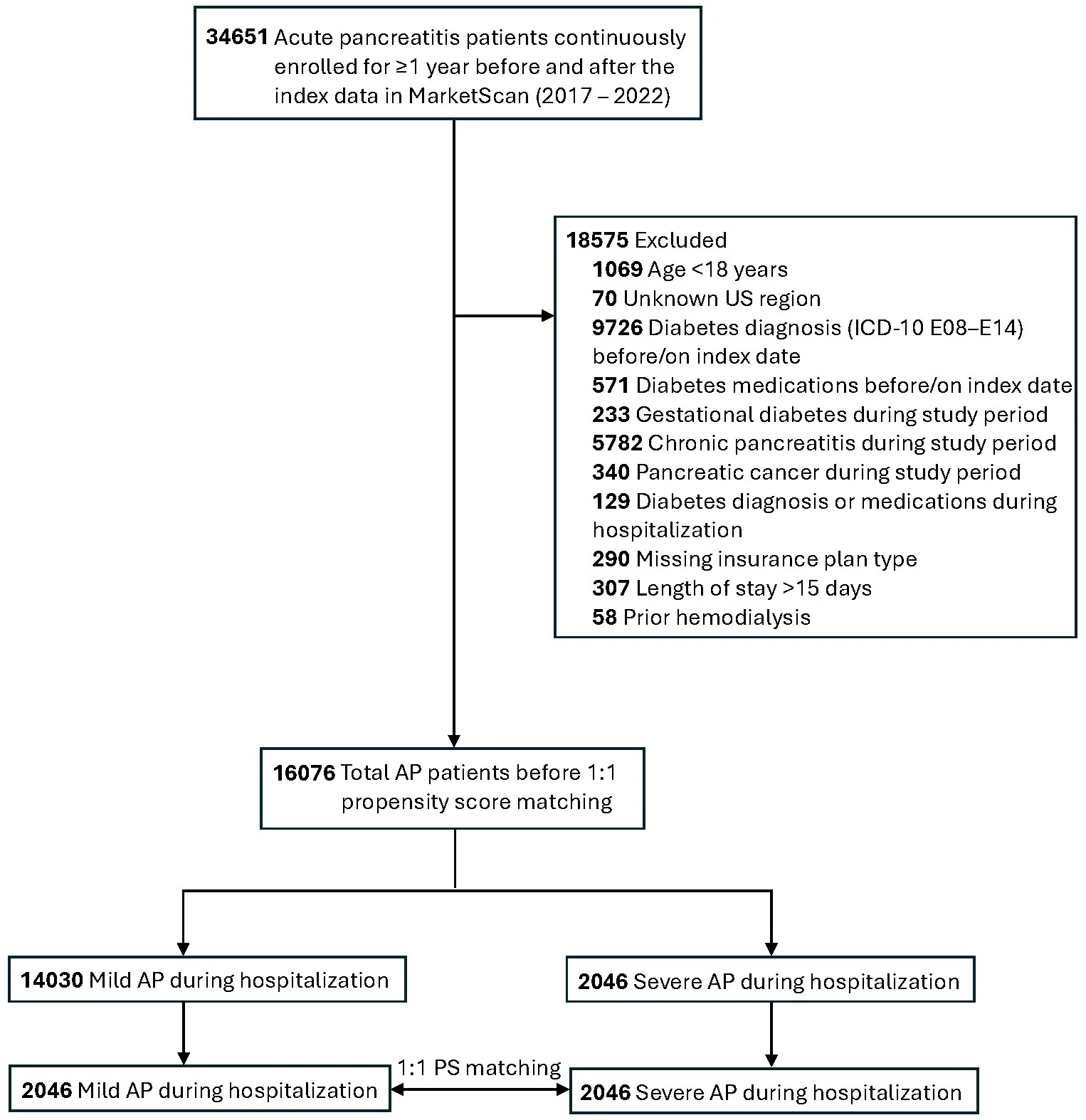
Flowchart of the study participants.

The study population included patients aged 18 to 64 years who were admitted to the hospital with a diagnosis of AP (International Classification of Diseases, Tenth Revision, Clinical Modification, ICD-10-CM code: K85) and were continuously enrolled for at least 12 months before the index admission date and at least 12 months after. The earliest inpatient admission diagnosis of AP in the database during the study period was defined as the index date of AP for each study participant. AP patients with prevalent diabetes or who were taking diabetes medication (**Supplementary Table 1**) before or on the index date were excluded from the study. Patients staying in the hospital longer due to primary or concurrent illnesses, which may inflate the rate of incident diabetes following hospitalization, were also excluded. To avoid confounding from severe comorbid diseases or nosocomial complications and reducing heterogeneity, we also excluded individuals with prolonged AP hospitalization (>15 days), gestational diabetes, chronic pancreatitis, and pancreatic cancer diagnosed during the study period. A complete list of exclusions is presented in the study population flow diagram (**Figure 1**). Data from 2017 to 2022 were primarily used for the study cohort derivation. The year prior to the index date was used to define baseline covariates, and 1 year following the index date ensured continuous insurance coverage. The overall study period covered 1 January 2016 to 31 December 2023. We conducted a propensity score matching using a greedy nearest neighbor approach in conjunction with a 1:1 matching ratio without replacement using the R package MatchIt.

The primary exposure of interest, severe AP, was defined using any of the following proxies in reference to the revised Atlanta classification during hospitalization: pancreatic necrosis, hemodialysis, shock, or organ failure, including renal, cardiac, respiratory, and mechanical ventilation, tracheal intubation, and vasopressor support (**Supplementary Table 2**). Mild AP was defined as the absence of all the above codes during the same period (4, 9).

### Assessment of outcomes

The main outcome of interest was the incidence of diabetes, defined as the presence of one of the following ICD-10 codes in the database during the follow-up period: E08–E14 (16–18). As in previous studies, E08, E09, and E14 are included to avoid missing incident cases of diabetes due to errors in coding (17–20).

### Assessment of covariates

Data on age (years), sex (men/women), and US region (South, West, North Central, Northeast) were extracted directly from the MarketScan database. Based on a comprehensive literature review, the following potential confounders, representing risk factors for AP and diabetes, were also captured using their corresponding ICD-10-CM, or Current Procedural Terminology (CPT) codes (**Supplementary Table 3**): obesity, hypertension, dyslipidemia, coronary artery disease (CAD), liver disease, alcohol abuse, smoking status, gallstones, number of office visits, use of glucocorticoids, statins, and antihypertensive medications, prediabetes, social determinants of health (SDOH), acute cholecystitis, chronic obstructive pulmonary disease (COPD), depression, and use of HIV-related medications (each, yes/no). The 12 months preceding the index date of AP were established as the baseline period.

### Statistical analysis

We performed univariable analysis to summarize the baseline characteristics of study participants in the mild versus severe AP groups using counts (percentages) for categorical variables and means (standard deviations) for continuous variables. These characteristics were presented both before and after 1:1 propensity score matching. Standardized mean difference (SMD) was used to assess covariate balance between the two groups, with an SMD greater than 0.1 indicating potential imbalance. Propensity scores were estimated from a logistic regression model using the covariates in the full model to balance baseline data between severe AP and mild AP. The goodness-of-fit of the multivariable logistic regression models was assessed using the Hosmer–Lemeshow test. A non-significant result (*P* > 0.05) indicates that the model fits the data well. Person‐time of follow-up for each participant was calculated from the index AP discharge date to the first occurrence of an outcome of interest, diabetes, maximum follow-up date (i.e., the latest date in the diagnosis records), end of enrollment, or end of the study period (31 December 2023), whichever took place first.

An initial Cox proportional hazards regression model was performed before propensity matching, adjusted for the aforementioned covariates. The final model was conducted using a stratified Cox proportional hazards regression model using matching IDs constructed from the propensity scores as the strata with the demographic, comorbidities, and medication factors, which provided adjusted hazard ratios (aHRs) and their 95% confidence interval (CIs). We performed several sensitivity analyses to test the robustness of our results. First, we analyzed to account for different follow-up periods. Second, the pathophysiology of alcoholic AP often differs from non-alcoholic AP. Therefore, we further excluded individuals with an alcoholic etiology of AP. Third, models including interactions with severe AP status, specifically age (years), sex, prediabetes, tobacco use, and alcohol abuse with new-onset diabetes risk, were assessed by the −2-log likelihood ratio (−2LL), stratified by the propensity score matching IDs. Subgroup analyses were further conducted following the interaction tests. Lastly, to assess how unmeasured confounding could have affected the observed association between severe AP and new-onset diabetes, we calculated the *E*-value using the methods of VanderWeele and Ding (21).


$$\text{If Estimate HR}>1,\text{ then E}-\text{value}=HR+\sqrt{HR*(HR-1)}$$

$$\text{If Lower Limit }(\text{LL})\text{ of CI}>1,\text{ then E}-\text{value}=LL+\sqrt{LL*(LL-1)}$$

A log–log survival curve was used to assess the violation of the proportional hazards (PHs) assumption. Data were analyzed using SAS Software version 9.4 (SAS Institute Inc., Cary, NC) and R software version 4.5.1 (R Foundation for Statistical Computing, Vienna, Austria) with a two-sided alpha level of 0.05.

## Results

In univariable analyses, individuals with severe AP were older, were more likely to be female, reside in the Southern US, and have a history of hypertension, CAD, liver disease, COPD, depression, alcohol abuse, tobacco use, and dyslipidemia. They were also more likely to use glucocorticoids, statins, and antihypertensive medications. However, after propensity score matching, baseline characteristics were well balanced between groups, as indicated by standardized mean differences (SMD < 0.1; **Table 1**; **Supplementary Figure 1**).

**Table 1:** Baseline characteristics before and after propensity score matching (PS) stratified by severe AP status.

Variables	Before 1:1 PS matching			After 1:1 PS matching		
	Mild AP*n* = 14,030	Severe AP*n* = 2,046	SMD^*^	Mild AP*n* = 2,046	Severe AP*n* = 2,046	SMD^a^
Demographics						
Age, mean (SD)	45.5 (12.3)	47.4 (12.0)	0.153	47.1 (11.7)	47.4 (12.0)	0.022
Female sex, *n* (%)	8,017 (57.1)	856 (41.8)	0.310	804 (39.3)	856 (41.8)	0.052
US region, *n* (%)			0.07			0.031
Northeast	2,252 (16.1)	280 (13.7)		267 (13.0)	280 (13.7)	
North Central	2,998 (21.4)	445 (21.7)		457 (22.3)	445 (21.7)	
South	6,694 (47.7)	992 (48.5)		1,009 (49.3)	992 (48.5)	
West	2,086 (14.9)	329 (16.1)		313 (15.3)	329 (16.1)	
Baseline comorbidities (yes)						
Prediabetes, *n* (%)	810 (5.8)	121 (5.9)	0.006	123 (6.0)	121 (5.9)	0.004
Obesity, *n* (%)	2,657 (18.9)	338 (16.5)	0.063	344 (16.8)	338 (16.5)	0.008
Hypertension, *n* (%)	4,543 (32.4)	890 (43.5)	0.231	855 (41.8)	890 (43.5)	0.035
CAD, *n* (%)	546 (3.9)	120 (5.9)	0.092	119 (5.8)	120 (5.9)	0.002
Liver disease, *n* (%)	1,808 (12.9)	308 (15.1)	0.063	286 (14.0)	308 (15.1)	0.031
COPD, *n* (%)	1,503 (10.7)	243 (11.9)	0.037	237 (11.6)	243 (11.9)	0.009
Acute cholecystitis, *n* (%)	331 (2.4)	29 (1.4)	0.069	21 (1.0)	29 (1.4)	0.036
Depression, *n* (%)	2,062 (14.7)	312 (15.2)	0.015	282 (13.8)	312 (15.2)	0.042
Dyslipidemia, *n* (%)	3,517 (25.1)	636 (31.1)	0.134	569 (27.8)	636 (31.1)	0.072
Tobacco use, *n* (%)	1,927 (13.7)	303 (14.8)	0.031	275 (13.4)	303 (14.8)	0.039
AP etiology/risk factor (yes)						
Alcohol abuse, *n* (%)	1,071 (7.6)	242 (11.8)	0.142	237 (11.6)	242 (11.8)	0.008
Gallstones, *n* (%)	2,367 (16.9)	219 (10.7)	0.180	217 (10.6)	219 (10.7)	0.003
Medications (yes)						
Glucocorticoids, *n* (%)	3,019 (21.5)	484 (23.7)	0.051	472 (23.1)	484 (23.7)	0.014
Statins, *n* (%)	1,691 (12.1)	366 (17.9)	0.164	333 (16.3)	366 (17.9)	0.043
Antihypertensives (%)	3,809 (27.1)	809 (39.5)	0.265	763 (37.3)	809 (39.5)	0.046
HIV drugs, *n* (%)	89 (0.6)	13 (0.6)	0.001	11 (0.5)	13 (0.6)	0.013
Healthcare utilization						
Office visits, mean (SD)	8.9 (11.0)	9.0 (11.5)	0.010	8.5 (10.7)	9.0 (11.5)	0.048
SDOH, *n* (%)	93 (0.7)	18 (0.9)	0.025	24 (1.2)	18 (0.9)	0.029

AP, acute pancreatitis; SMD, standardized mean difference; SD, standard deviation; CAD, coronary artery disease; COPD, chronic obstructive pulmonary disease; HIV, human immunodeficiency virus; SDOH, social determinants of health.

^*^ All covariates included in the model presented standardized mean differences <0.1, indicating that groups were well balanced after propensity score matching.

The Hosmer–Lemeshow test yielded a *P*-value of 0.86, which confirmed that the models fit the data well. There was no evidence of violation of the proportional hazards assumption (PHA) (*P* = 0.45). Additionally, log–log survival curves for checking the PHA for the two groups were parallel, suggesting no violation (**Supplementary Figure 2**).

**Figure 2:**
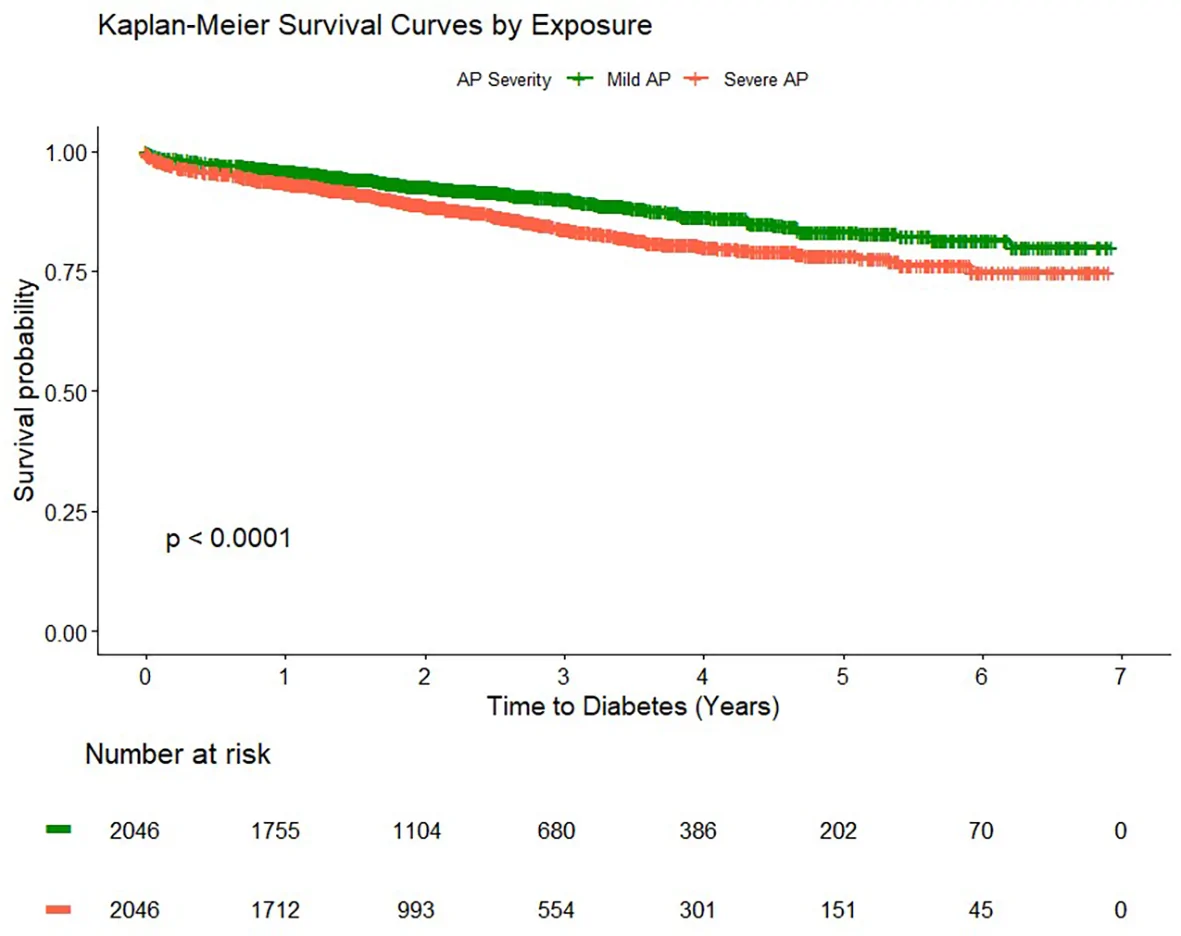
Kaplan–Meier survival curve.

The overall unadjusted cumulative incidence and incidence density rate of diabetes were higher among participants with severe AP (56.6 per 1,000 person-years) compared to those with mild AP (36.8 per 1,000 person-years) (**Table 2**; **Figure 2**). In the stratified multivariable Cox regression model by the propensity score matching strata, severe AP was associated with a higher risk of incident diabetes (aHR = 1.64, 95% CI: 1.30–2.06) (**Table 2**). The association was stronger after excluding those with alcohol-related AP (aHR = 1.67, 95% CI: 1.26–2.22) (**Table 2**). Suggesting a strong early effect, severe AP was associated with a two-fold increased risk of diabetes within 90 days following discharge (aHR = 2.06, 95% CI: 1.36–3.12). Similar effects were observed at 6 months and 3 years of follow-up, with some attenuation over time.

**Table 2:** Cox proportional hazards models showing hazard ratios (HRs) and 95% confidence intervals (CIs) before and after PSM, assessing the association between severe AP during hospitalization and incident diabetes mellitus.

Variable	Mild AP	Severe AP
New onset diabetes cases, *n*	190	268
Person-years (PY), *n*	5,167	4,736
*Incidence rate, 95% CI per 1,000 PY	36.8 (31.9, 42.4)	56.6 (50.2, 63.8)
§Model 1^b^	(Reference)	1.60 (1.39, 1.83)
Model 2^c^	(Reference)	1.64 (1.30, 2.06)
Sensitivity analyses^a^		
Exclude patients with alcoholic AP (K85.2)	(Reference)	1.67 (1.26, 2.22)
90 days of follow-up	(Reference)	2.06 (1.36, 3.12)
6 months of follow-up	(Reference)	1.75 (1.24, 2.46)
1 year of follow-up	(Reference)	1.70 (1.28, 2.27)
3 years of follow-up	(Reference)	1.67 (1.32, 2.11)
5 years of follow-up	(Reference)	1.64 (1.30, 2.07)

PSM, propensity score matching.

^*^ Unadjusted incidence rate per 1,000 person-years.

^§^ Model 1: The fully adjusted Cox proportional hazards model before PSM included the following covariates: age, sex, US region, obesity, hypertension, dyslipidemia, coronary artery disease (CAD), liver disease, alcohol abuse, smoking status, gallstones, number of office visits, use of glucocorticoids, statins, and antihypertensive medications, prediabetes, social determinants of health (SDOH), acute cholecystitis, chronic obstructive pulmonary disease (COPD), depression, and use of HIV-related medications.

Model 2: Stratified by the fully matched propensity score matching strata, using the covariates listed in model 1.^a^Analyses based on the full.Stratified by the fully matched propensity score matching strata.

In comparing the modifying effect of patient demographics and comorbidities, a stronger association between severe AP and diabetes was observed in men (aHR = 2.03, 95% CI: 1.50–2.74) than in women (aHR = 1.06, 95% CI: 0.69–1.64; *P*_interaction_ = 0.02; **Supplementary Figure 3**). No significant interactions between severe AP and diabetes were found for age, prediabetes, tobacco use, and alcohol abuse (*P*_interaction_ > 0.05 for all; **Supplementary Figure 3**). An *E*-value of 2.66 with a lower confidence limit (LL) of 1.92 indicates that unmeasured confounders in the MarketScan^®^ database, such as race/ethnicity or imaging-related biomarkers, are unlikely to fully account for the observed association between severe AP and new-onset diabetes.

## Discussion

In this real-world evidence study involving more than four thousands of AP patients with no prior history of diabetes, we found that patients with severe AP had a higher incidence rate of diabetes compared to those with mild AP (56.6 per versus 36.0 per 1,000 person-years). Adjusted analyses demonstrated a two-fold increased risk of diabetes within 90 days following hospital discharge. The risk remained elevated, albeit to a smaller degree, for up to 3 years of follow-up. The robustness of our findings was illustrated using propensity score matching and was independent of demographics, major chronic medical conditions, and medications. To our knowledge, this is the first largest study using causal inference and time-to-event approaches to evaluate the association between severe AP during hospitalization and the risk of new-onset diabetes using real-world data from a large, insured US population.

While the risk of developing diabetes is higher in the first months after AP, it does not appear to return to the risk of the general population, maintaining a cumulative incidence reported to be as high as 40% (22, 23). The resolution of stress hyperglycemia following an episode of AP is not quantified. However, one study showed that 95.8% and 68.4% of patients with mild and severe AP had normalization of serum glucose after pancreatitis treatment (24). In contrast, concurrent clinical conditions that affect the red blood cell turnover, as in the case of hemolytic anemia or blood loss, may result in falsely low HbA1c values regardless of glycemic status (25–27).

The findings of this study have both public health and clinical implications, emphasizing the importance of surveillance and screening after hospitalization, especially among high-risk patients after an episode of severe AP (28). Monitoring for dysglycemia and early intervention may prevent or slow progression to diabetes, with the ultimate goal of preventing future complications. Routine monitoring of diabetes-related biomarkers, including fasting glucose and HbA1c, is recommended within 3 months after discharge from severe AP (29). Ideally, continuity of care after discharge may also include multidisciplinary care with endocrinologists, gastroenterologists, surgeons, and dietitians/nutritionists, as required. These integrated services would focus on risk reduction tailored to pancreatitis etiology, surveillance, nutritional optimization, care of complications, and coordinated follow-up.

Our findings are consistent with previous studies that have investigated the association between severe AP using the 2012 revised Atlanta classification criteria (4, 23, 30–32). A recent meta-analysis including 50 studies found that severe AP was associated with significantly increased odds of developing diabetes (OR: 1.86; 95% CI: 1.27, 2.73) (23). Furthermore, another study from our group using the Nationwide Readmission Database found that severe AP was independently associated with higher odds of AP-related diabetes (4).

The plausible biological mechanisms for the observed association between severe AP and the development of incident diabetes may include beta-cell death, as in severe cases of AP and persistent organ failure, or islet cell destruction in the affected part of the pancreas caused by inflammation or pancreatitis-related necrosis (23, 30, 33). In addition to loss of beta cells resulting from pancreatic necrosis or resection, systemic inflammation may also promote insulin resistance and impair islet function. Furthermore, exocrine pancreatic insufficiency disrupts incretin signaling, reducing insulin secretion and worsening hyperglycemia (6). Islet cell autoimmunity appears to develop in a subset of patients, although the mechanism remains unknown (34). Lastly, there are likely overlapping and shared risk factors, including obesity and genetic predisposition to the development of hyperglycemia. Collectively, diabetes following AP is likely multifactorial in nature, related to islet cell destruction as well as a consequence of genetic predisposition, systemic and local inflammation, and hormonal dysregulation.

Consistent with prior findings, the relationship between severe AP and new-onset diabetes differed significantly between men and women, with a stronger association observed in men (35). This variation may reflect underlying biological and hormonal influences. Differences in hormone activity affecting pancreatic beta-cell function, body fat distribution, and metabolic regulation likely contribute. Men generally exhibit greater central adiposity, in contrast to women who preferentially store fat subcutaneously. Furthermore, greater insulin resistance in men can amplify pancreatic inflammation and metabolic dysfunction following AP. In addition, lifestyle and comorbid factors such as alcohol use, hypertension, and obesity may further elevate diabetes risk in men (36). These findings highlight the need to account for potential biological and behavioral differences between men and women when developing surveillance and diabetes prevention strategies after AP.

### Study strengths and limitations

The strengths of our study include the use of propensity score matching combined with time-to-event analysis of longitudinal data from a large cohort of patients with AP. This approach accounts for both timing and censoring of events, offering advantages over traditional logistic regression models that do not consider these factors. While some data fields lack precision in this dataset, such as imaging-related biomarkers, our study’s relatively high *E*-values indicate robustness against potential unmeasured confounding. Nonetheless, our study has several limitations that should be considered when interpreting the results. This observational study used medical claims data; therefore, causality cannot be established. In addition, our study included only AP patients who had continuous enrollment in their private insurance plan from 12 months before to 12 months after the index AP date, which may introduce some selection bias. However, continuous enrollment was necessary to understand the contribution of pre-existing illnesses and ensure sufficient follow-up for the key outcome. We also acknowledge that claims-based databases may misclassify patients due to misreporting or underreporting of diagnoses or medications. Additionally, the MarketScan database does not include data on race or ethnicity, income, laboratory results, or imaging-based biomarkers. Therefore, we could not directly assess severity based on the extent of imaging abnormalities or pancreatic morphological characteristics (e.g., fat infiltration and fibrosis). There is an ongoing prospective cohort study of AP participants that is obtaining a comprehensive characterization of laboratory, metabolic, and imaging parameters to determine their contributions to incident diabetes (37–39). Finally, MarketScan includes few, if any, individuals aged 65 years. Therefore, while we cannot think of biological reasons why this would be the case, our findings may not be generalizable to older adults with AP and those without health insurance. Future studies could address this gap in the literature by using alternative real-world databases.

## Conclusions

In this large US real-world study, we found that severe AP was associated with increased risk of incident diabetes. The association between the severity of AP and diabetes was two-fold higher within 90 days following hospital discharge. These findings highlight the importance for glycemic surveillance and the need to consider proactive management of severe AP patients to mitigate their risk of poor health outcomes.

## Data Availability

The data analyzed in this study is subject to the following licenses/restrictions: The data used in this study were obtained from a third party, Merative MarketScan, although access is restricted because they were used under a Penn State College of Medicine license for this study and are not publicly available. The data can be accessed from Merative (https://www.merative.com/products/marketscan-research-databases). Requests to access these datasets should be directed to https://www.merative.com/products/marketscan-research-databases.
