# Supplementary material for: Association between severe acute pancreatitis and new-onset diabetes: a propensity score-matched real-world study
Source: Front Endocrinol (Lausanne). 2025 Nov 14;16:1704688. doi: 10.3389/fendo.2025.1704688 (PMC12660104; doi:10.3389/fendo.2025.1704688)

Supplemental Tables: Lifestyle factors, clinical Conditions, and diabetes medications.

Supplemental Table 1| Diabetes medications by class and generic name.

Medication Class	Generic Names
Biguanides	Metformin
Insulin	Insulin
Sulfonylureas	Glipizide, Glyburide, Gliclazide, Glimepiride
Meglitinides	Repaglinide, Nateglinide
DPP-4 Inhibitors	Sitagliptin, Linagliptin, Saxagliptin, Vildagliptin, Alogliptin
GLP-1 Receptor Agonists	Liraglutide, Semaglutide, Dulaglutide, Exenatide, Lixisenatide, Albiglutide
SGLT2 Inhibitors	Canagliflozin, Empagliflozin, Dapagliflozin, Ertugliflozin
Thiazolidinediones (TZDs)	Pioglitazone, Rosiglitazone
Alpha-Glucosidase Inhibitors	Acarbose, Miglitol
Amylin Analogues	Pramlintide
Dopamine Agonists	Bromocriptine
Bile Acid Sequestrants	Colesevelam

Supplemental Table 2| ICD-10, ICD-10-PCS, and procedure codes (CPT) used to identify severe AP during hospitalization.

Condition / Procedure	ICD-10, ICD-PCS / CPT codes
Pancreatic Necrosis	K85.91, K85.92
Hemodialysis	CPT codes: 90935, 90937, 90945, 90947, 90999; ICD-10-PCS: 5A1D00Z, 5A1D60Z
Shock	R57
Acute and Unspecified Kidney Failure	N17, N19
Cardiac Failure	I50.1–I50.4, I50.9; ICD-10-PCS: 3E033XZ
Acute Respiratory Failure and Mechanical Ventilation	J96.0–J96.9, Z99.11, R09.2; ICD-10-PCS Codes: 5A1935Z, 5A1945Z, 5A1955Z, 0BH17EZ; CPT codes: 31500, 96365, 96366

Supplemental Table 3| ICD-10 and procedure codes (CPT) used to identify baseline covariates/clinical conditions, and lifestyle factors.

**Condition**	**ICD-10 / CPT codes**
Obesity	E66
Hypertension	I10–I15
Dyslipidemia	E78
Coronary Artery Disease (CAD)	I20–I25
Liver Disease	K70–K77
Alcohol abuse	F10, K70, T51, G31.2, G62.1, G72.1, I42.6, K29.2, Y91.2, Y91.3
Tobacco use	F17, Z71.6, Z72.0, T65.2, O99.33, Z87.891, CPT-codes: 99406, 99407, G0375, G0376, G0436, G0437, G9016, G9276, G9458, G8402, G8403, G8453, G8454, S4990, S4991, S4995, S9075, S9453, 4000F, 4001F
Gallstones	K80
Social Determinants of Health (SDOH)	Z55–Z65
Acute Cholecystitis	K81.0
Chronic Obstructive Pulmonary Disease (COPD)	J40–J45, J47, J44.9
Depression	F32, F33, F34.1, F43.21, F32.9
Prediabetes	R73.01, R73.02, R73.03, R73.09

Supplementary Figure 1: Balance of covariates before and after propensity score matching.

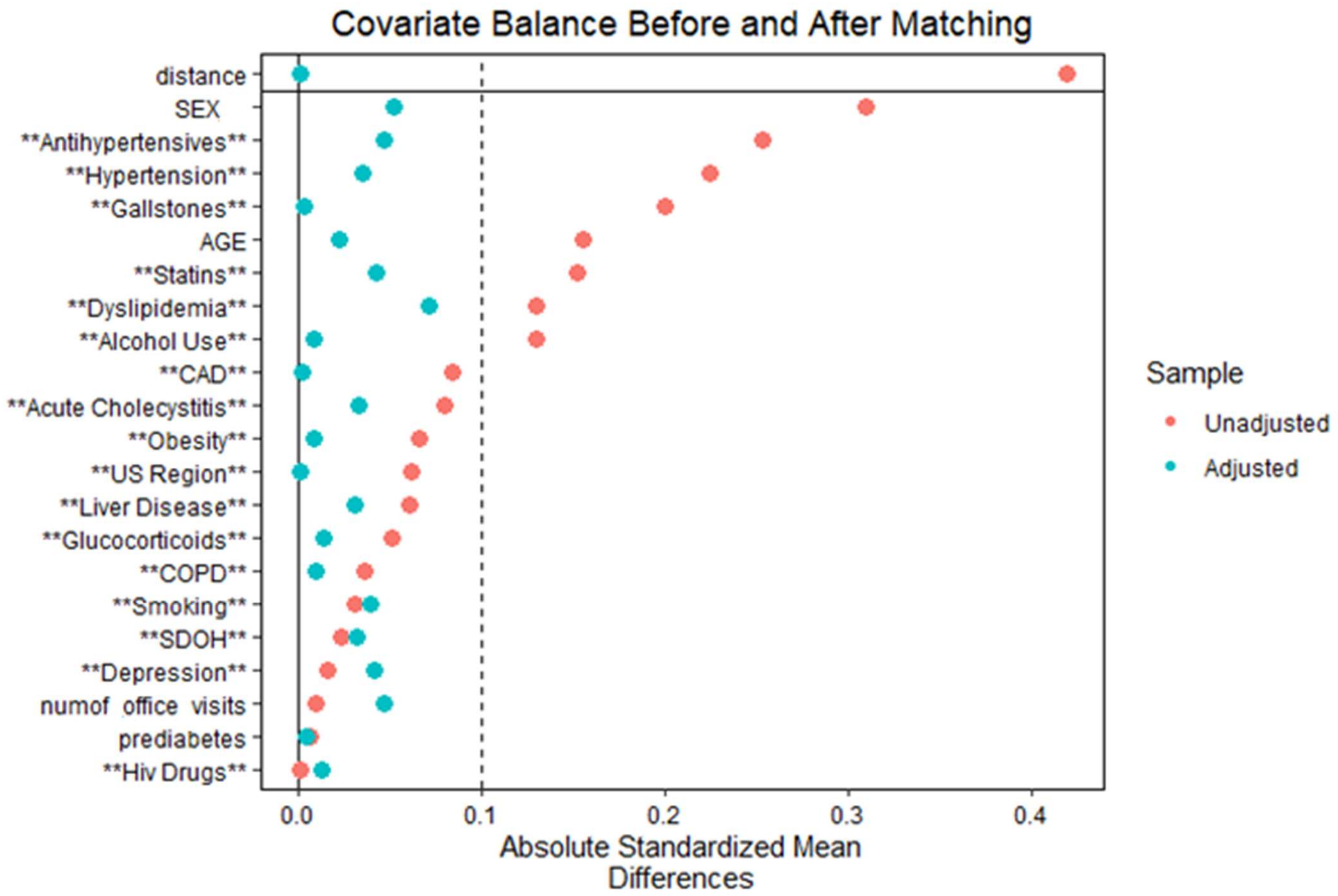

Supplementary Figure 2: Visually assessing the proportional hazards assumption.

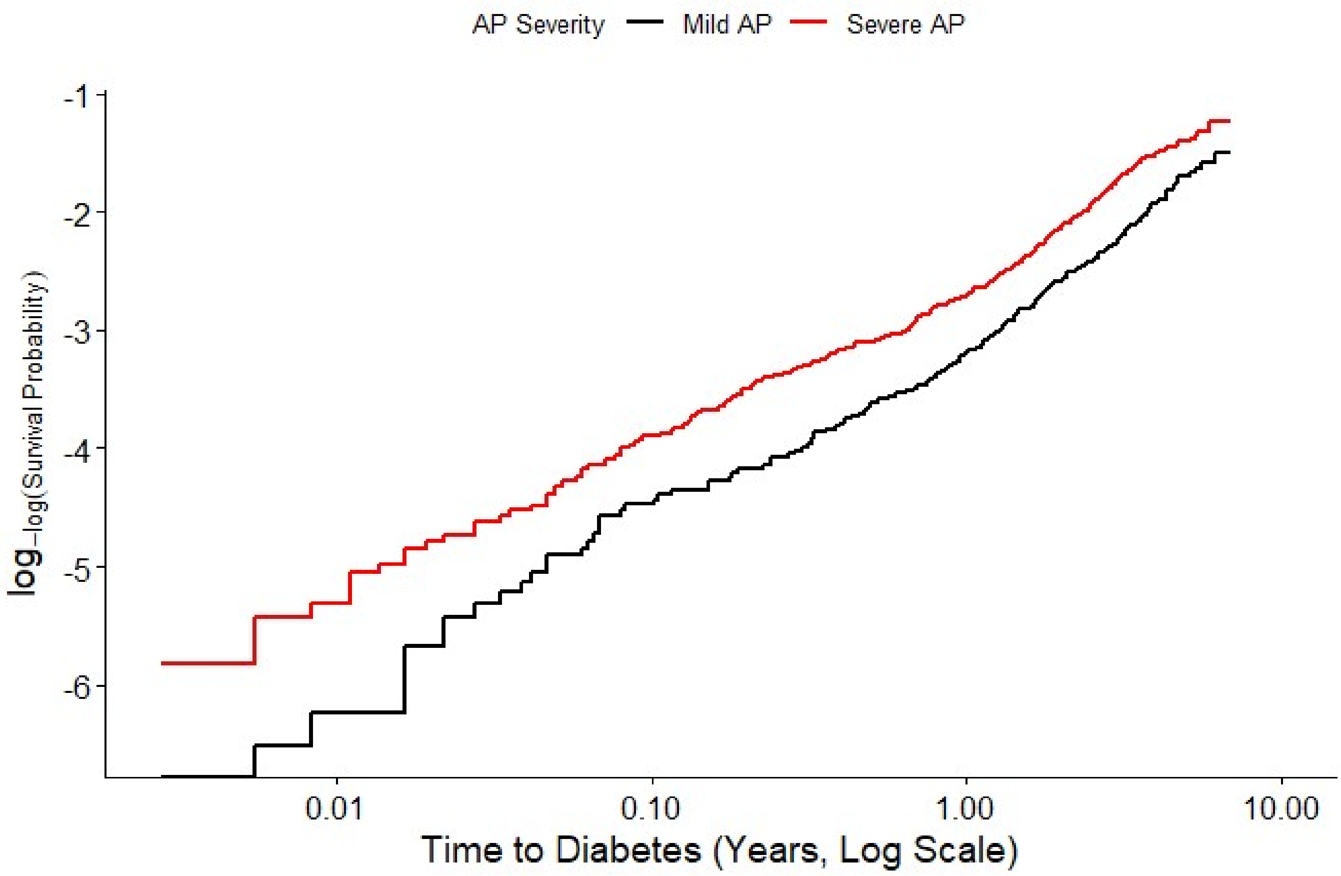

Supplementary Figure 3: Subgroup analysis showing the HRs stratified by age group, sex, prediabetes, tobacco use, and alcohol abuse.

Category	Subgroup	HR	95% CI	*P* for interaction
Age Group	18–40	1.33	(0.67, 2.64)	0.49
	41–64	1.77	(1.34, 2.33)	
Sex	Men	2.03	(1.50, 2.74)	0.02
	Women	1.06	(0.69, 1.64)	
Prediabetes	No	1.62	(1.27, 2.07)	0.61
	Yes	2.34	(0.60, 9.07)	
Tobacco use	No	1.53	(1.18, 1.99)	0.33
	Yes	2.42	(1.06, 5.51)	
Alcohol abuse	No	1.56	(1.20, 2.02)	0.44
	Yes	2.14	(1.03, 4.47)	

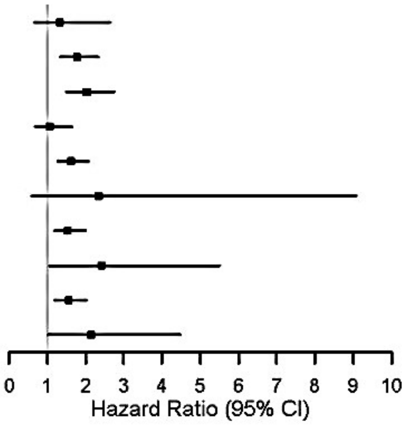

Supplement: Supplementary file 1 [file DataSheet1.pdf]
